# Attending an integrated nephrology and diabetology outpatient service can improve diabetic kidney disease treatment: a single-center experience

**DOI:** 10.1007/s00592-024-02423-w

**Published:** 2025-02-21

**Authors:** S. Wolde Sellasie, C. Pecchioli, K. Cersosimo, I. Nardone, S. Zaccaria, A. Centi, P. Di Perna, P. Tatangelo, P. Sperti, G. Schifano, L. Giurato, A. Bellia, R. Palumbo, L. Uccioli

**Affiliations:** 1 Division of Endocrinology and Diabetes, Department of Biomedicine and Prevention, CTO Andrea Alesini Hospital, University of Rome Tor Vergata, 00133 Rome, Italy; 2 PhD School of Applied Medical-Surgical Sciences, University of Rome Tor Vergata, 00133 Rome, Italy; 3 Unit of Nephrology, Sant’ Eugenio Hospital, 00144 Rome, Italy; 4 Department of Systems Medicine, University of Rome “Tor Vergata”, Via Montpellier 1, 00133 Rome, Italy; 5 School of Specialization in Food Sciences, University of Rome Tor Vergata, Via Montpellier 1, 00133 Rome, Italy

**Keywords:** Diabetic nephropathy, Diabetic kidney disease, Chronic kidney disease, SGLT-2 inhibitors

## Abstract

**Background:**

Glucose-lowering medications with established reno-protective effects are still underused in Italy. We explored whether attending an integrated nephrology and diabetology (NPD) outpatient service can improve clinical outcomes and adherence to treatment guidelines for diabetic kidney disease (DKD).

**Methods:**

We retrospectively included 110 DKD patients (aged 71.1 ± 10.1 years; 74.5% males) having attended the NPD outpatient service of CTO Hospital (Rome) between June and November 2023. Age- and gender-matched control group included DKD patients attending regular Diabetology outpatient service. Drugs prescriptions, clinical and biochemical parameters related to routine evaluation of DKD were collected at first and 6-months control visit.

**Results:**

This DKD population was made of 28.2% of patients with urine albumin-creatinine ratio (UACR) > 30 mg/gr, 33.6% with glomerular filtration rate (GFR) < 60 ml/min, 38.2% with both abnormalities. Proportion of patients prescribed with most recent anti-diabetic medications significantly increased after attending the NPD service (for SGLT-2 inhibitors, 54.5 vs. 25.5%, *p* < 0.01; for GLP1-R agonists, 28.3 vs. 21.8%, *p* = 0.01), as well as for statins (*p* < 0.01) and calcium channel-blockers (*p* = 0.01). During the same observation period we registered significant reduction in LDL cholesterol (*p* = 0.01) and UACR levels (*p* = 0.007), with a trend toward improvement in HbA1c and eGFR. Conversely, no significant differences in drugs prescriptions were reported in the control group, except for SGLT-2 inhibitors.

**Conclusions:**

Enhanced real-time interaction and collaborative decision-making in an outpatient setting that integrates both diabetology and nephrology expertise can lead to better clinical outcomes and greater adherence to DKD management guidelines, ultimately providing a more comprehensive strategy for cardio-renal risk reduction.

**Supplementary Information:**

The online version contains supplementary material available at 10.1007/s00592-024-02423-w.

## Introduction

Renal damage is one of the most challenging chronic complications of diabetes mellitus (DM) [1], posing a significant risk of accelerated cardiovascular disease and progression to end-stage renal disease (ESRD) [2]. Previous epidemiological studies have highlighted the natural history and heterogeneity of this complication, leading to the adoption of the term “diabetes kidney disease” (DKD) to encompass all types of renal damage in DM [3]. According to the Global Burden of Diseases Study 2019, over the period from 1990 to 2019, there has been a substantial increase of 150% and 120% in overall mortality and disability-adjusted life years (DALYs) related to DKD, respectively [4]. Severity of these outcomes is particularly noteworthy in advanced stages of DKD, often culminating in ESRD with the need for haemodialysis [5]. DKD may be initially recognized because of detection of microalbuminuria and/or renal impairment, progressing towards ESRD through pathways characterized by either increasing albuminuria or decreasing glomerular filtration rate (GFR) [6]. In addition, both albuminuria and reduced glomerular filtration rate are associated with an increased risk of cardiovascular events and mortality [7]. For decades, management strategies to curb DKD progression have solely involved optimization of glycemic control and the use of blood pressure-lowering medications, particularly angiotensin-converting enzyme (ACE) inhibitors and angiotensin II receptor blockers (ARBs), which are also effective in reducing albumin excretion [8]. More recently, new classes of anti-hyperglycemic drugs, such as sodium-glucose cotransporter type 2 inhibitors (SGLT-2 inhibitors) and glucagon-like peptide 1 receptor agonists (GLP1-R agonists), have been shown to reduce both the progression of albuminuria and the decline of GFR in patients with diabetes and high cardiovascular risk, even on top of available anti-albuminuric agents [9–11].

Despite the robust evidence supporting efficacy of these new classes of drugs also in the managing of DKD, optimal care of these patients is not consistently achieved in clinical practice. According to the 2022 Associations of Medical Diabetologists (AMD) Annals data in Italy, among subjects with DM and high levels of albuminuria, 47.2% were not treated either with SGLT-2 inhibitors or GLP1-R agonists, whereas among subjects with GFR below 60 ml/min, 73.4% of them were not prescribed with SGLT-2 inhibitors [12]. This observed under-prescription of anti-diabetic drugs with proven renal benefits can be attributed to a number of reasons including therapeutic inertia, concerns about potential side effects, inadequate patient’s follow-up, and also the lack of communication between nephrologists, diabetologists, and other medical specialties [13].

Treatment approach to DKD could be optimized through a multidisciplinary team framework, similar to strategies used for other chronic diabetes-related complications [14]. An integrated approach could enhance the confidence of specialists in managing and selecting optimal therapeutic options, thereby helping overcome clinical inertia and ensuring alignment with current treatment guidelines [1, 15–17]. According to this background, here we report the initial experience of a single outpatient service in Italy that employs an integrated diabetology/nephrology approach, in terms of adherence to current treatment recommendations for patients with DKD.

## Methods

### Study design and participants

We retrospectively reviewed the electronic clinical records of patients attending the integrated nephrology and diabetology (NPD) outpatient service of the CTO-Alesini University Hospital (Rome, Italy) during the period June-November 2023. This integrated outpatient service has been implemented through the collaboration of the Endocrinology and Nephrology Divisions of CTO-Alesini Hospital, with the goal of improving the care of diabetic patients with concomitant DKD according to current management guidelines. Briefly, patients are referred for the first time to the NPD service by the regular Diabetology or Nephrology outpatient service if diagnosed with diabetes mellitus and with evidence of albuminuria and/or impaired renal function. At first NPD visit, the patient is simultaneously visited by a diabetologist and a nephrologist, who provide the patient with medications prescriptions and planning of subsequent exams and follow-up NPD visits.

Inclusion criteria for the present analysis were as follows: (1) age > 18 years; (2) diagnosis of DM; (3) presence of CKD with eGFR < 60 ml/min (according to CKD-EPI formula) and/or urine albumin-creatinine ratio (UACR) > 30 mg/g in at least two repeated measurements [18]; (4) availability of data on HbA1c, eGFR, UACR, LDL-C, and about ongoing anti-hyperglycemic and other DKD-related medications at the time of the first NPD visit (index visit) and after six months of follow-up (follow-up visit). Patients on hemodialysis were not included.

Age- and gender-matched control group was extracted among type 2 diabetic patients with evidence of DKD (presence of albuminuria and/or impaired renal function) who had attended the regular Diabetology outpatient service in the year before implementation of the NPD service at our University Hospital, and for whom complete clinical and drugs prescriptions data were available. As described before for the NPD group, we retrospectively collected control group data at the first visit (index visit) and after six months of follow-up (follow-up visit).

All subjects were adult white Europeans and gave their own written informed consent to be included in the analysis. The study protocol conformed to the ethical guidelines of the 1975 Declaration of Helsinki and was approved by the local Ethic Committee.

## Data collection and outcomes

For both study and control group, data were extracted using the electronic clinical records regularly provided by the hospital outpatient service. The following clinical and demographic data were collected at the first visit (index visit): age, gender, body mass index (BMI; calculated as weight in kilograms divided by height in meters square), systolic and diastolic blood pressure, duration of diabetes (years), previous nephrology evaluation (yes/no). Biochemical data including creatinine, eGFR (according to CKD-EPI formula), UACR, urate, glycated hemoglobin (HbA1c), fasting glucose, total cholesterol, HDL-C, LDL-C, triglycerides were collected at the index visit and at the follow-up visit after six months. Prescriptions of medications were similarly captured at the index visit and at the follow-up visit from the electronic records.

### Statistical analysis

Statistical analysis was performed using the SPSS 25.0 software (SPSS, Chicago). Mean ± SD or median (interquartile range) were used as descriptive statistics for normally distributed or skewed continuous variables, respectively. Categorical variables were expressed as absolute and percent values. All quantitative variables were tested for normality distribution using the Kolmogorov-Smirnov test and continuous parameters with a non-normal distribution were logarithmically transformed before being used in the subsequent parametric procedures. The study population was stratified into three subgroups according to DKD phenotype: (1) presence of UACR > 30 mg/gr creatinine with eGFR > 60 ml/min (ALB group); (2) presence of eGFR < 60 ml/min with normal UACR (CKD group) and (3) presence of both UACR > 30 mg/gr creatinine and eGFR < 60 ml/min (ALB + CKD group). Differences between the three identified subgroups in quantitative variables were assessed using the analysis of variance (ANOVA) test, with the subsequent Bonferroni test for post-hoc analysis. Differences in qualitative variables were assessed with the chi-square test. Patients prescribed with individual classes of anti-hyperglycemic and other selected medications were given as absolute and percent values. Differences in prescriptions for all the selected classes of drugs between index and follow-up visits were compared using the McNemar test. Differences in eGFR, LDL-C, UACR and HbA1c, between index and follow-up visit were assessed using Student’s *t* test for paired data. For all these analysis a *p*-value < 0.05 based on a two-tailed statistical test was considered as significant.

## Results

Overall 110 patients with DKD were included, the majority of whom had type 2 diabetes. Median age was 72 years and 74.5% were men. Median duration of DM was 11 years, with a mean glycated hemoglobin of 7.5% (58 mmol/mol). The majority of patients (76.4%) had never received a nephrology evaluation before. When stratified according to DKD subtypes, 28% of subjects had only UACR > 30 mg/gr (ALB group), 34% had only eGFR < 60 ml/min (CKD group), whereas 38% had both conditions (ALB + CKD). Of interest, only 12.9% of patients in the ALB group had been previously referred to a nephrology evaluation, compared with patients in the ALB + CKD group (35.8%) (Table 1). No significant differences between groups were observed in glycemic control, BMI, blood pressure and lipid profile.

**Table 1:** Main clinical characteristics of the study group stratified by DKD subtypes

	Total (*n* = 110)	ALB (*n* = 31)	CKD (*n* = 37)	ALB + CKD (*n* = 42)	*p*-value^¶^
Gender					0.165
*Female*	28 (25.5%)	4 (12.9%)	11 (29.7%)	12 (31%)	
*Male*	82 (74.5%)	27 (87.1%)	26 (70.3%)	29 (69%)	
Age (years)	71.1 ± 10.2	63.9 ± 8.7 ^† ‡^	76.9 ± 8 ^§^	71.5 ± 9.4	< 0.01
BMI*	28.7 (25.1–31.5)	28.3 (25.9–31.5)	28.7 (24.9–30.6)	28.7 (24.9–33.2)	0.51
DM duration (years)*	11 (5-19.5)	10 (5–15)	12 (4–18)	12.5 (6–20)	0.39
Previous nephrology evaluation					0.003
*Yes*	26 (23.6%)	4 (12.9%) ^‡^	7 (18.9%)	15 (35.8%)	
*No*	84 (76.4%)	27 (87.1%)	30 (81.1%)	27 (64.2%)	
Creatinine (mg/dL)*	1.6 (1.3-2.0)	0.9 (0.8–1.1) ^† ‡^	1.8 (1.5–2.1)	1.8 (1.6–2.3)	< 0.01
eGFR CKD-EPI (ml/min)*	40.3 (29-60.5)	83.6 (67.3–95.7) ^† ‡^	33 (27-40.8)	36.1 (27.1–42)	< 0.01
UACR (mg/gr)*	91 (29–338)	142 (69–316) ^† ‡^	5 (3-12.3) ^§^	271 (54–706)	< 0.01
Urate (mg/dL)	6.3 ± 1.8	6.1 ± 2	6.5 ± 2	6.3 ± 1.4	0.71
Glycated Haemoglobin % (mmol/mol)*	7.1(6.5–8.3) 54 (47–67)	6.9(6.4–8.2) 52 (46–66)	7.0(6.5–7.5) 53 (48–59)	7.3(6.6–8.1) 56 (49–65)	0.25
Glucose (mg/dL)	142 ± 57	152 ± 65	135 ± 34	142 ± 40	0.37
LDL-Cholesterol (mg/dL)	88 ± 37	87 ± 52	85 ± 48	92 ± 50	0.75
Triglycerides (mg/dL)*	125 (93–188)	120 (92–167)	144 (96–202)	138 (98–200)	0.54
Systolic blood pressure (mmHg)	136 ± 18	135 ± 18	135 ± 20	139 ± 17	0.55
Diastolic blood pressure (mmHg)	75 ± 10	71 ± 9	72 ± 10	75 ± 10	0.48

Data expressed as means ± SD, medians (i.q. range) or proportions (percentage) as appropriate

^¶^ANOVA test for continuous variables or chi-square test for discrete variables, as appropriate

*Log-transformed before performing parametric tests. †=post hoc ALB vs. CKD with *p* < 0.05

^‡^Post hoc ALB vs. ALB + CKD with *p* < 0.05

^§^Post hoc CKD vs. ALB + CKD with *p* < 0.05. Abbreviations as described in the text

In Table 2 we reported proportions of patients (absolute and percent values) prescribed with different classes of anti-hyperglycemic medications, at index and follow-up visit. Of note, insulin was the most prescribed medication (44.5%) before attending the NPD service, followed by metformin (30.9%), SGLT-2 inhibitors (25.5%) and GLP1-R agonists (21.8%). Only 11.8% of patients were taking sulfonylureas. Of note, when we restricted the analysis to the small subgroup of patients (*n* = 26) who had already received a previous nephrology evaluation, prescriptions of SGLT-2 inhibitors were significantly higher (*p* < 0.01) compared to those who had never been visited by a nephrology specialist. Among other DKD-related medications, statin therapy was reported by 58.2% of patients before the visit, whereas the most prescribed anti-hypertensive classes of drugs were calcium channel blockers (41.8%), ACE-inhibitors (35.5%) and angiotensin-receptor blockers (ARBs) (35.5%). After six months from the initial NPD visit, we registered an increase in prescriptions of anti-hyperglycemic drugs with known reno-protective effects (for SGLT-2 inhibitors 25.5 vs. 54.5%, *p* < 0.01; for GLP-1R agonists 21.8 vs. 28.3%, *p* = 0.01), alongside a substantially increased number of patients taking statins (58.2 vs. 78.1%, *p* < 0.01) and calcium channel blockers (41.8 vs. 48.1%, *p* = 0.01) (Table 2). When comparing several clinical outcomes between the index and follow-up visit, we observed significant improvement in UACR [91 (29–338) vs. 77 (17–260) mg/gr; *p* = 0.007] and LDL-C (88 ± 37 vs. 88 ± 37 mg/dl, *p* = 0.01), whereas eGFR [40 (29–60) vs. 44 (33–62) ml/min] and HbA1c [54 (47–67) vs. 52 (44–65) mmol/mol] showed only a non-significant trend toward improvement.

**Table 2:** Clinical outcomes and proportion of patients prescribed with different anti-hyperglycemic agents and other DKD-related medications before and after attending the NPD outpatient service

	At NPD visit	After 6 months from NPD visit	*p*-value^a^
Glycated haemoglobin^b^			
%	7.1 (6.5–8.3)	6.9 (6.1–8.1)	0.14
(mmol/mol)	54 (47–67)	52 (44–65)	
eGFR CKD-EPI (ml/min)^b^	40 (29–60)	44 (33–62)	0.09
LDL-Cholesterol (mg/dL)	88 ± 37	79 ± 35	0.01
UACR (mg/gr)^b^	91 (29–338)	77 (17–260)	0.007
SGLT-2 inhibitors	28 (25.5%)	59 (54.5%)	< 0.01
GLP1-R agonists	24 (21.8%)	33 (28.3%)	0.01
Insulin	49 (44.5%)	54 (49.1%)	0.06
Metformin	34 (30.9%)	31 (28.2 %)	0.40
DPP-IV inhibitors	19 (17.3%)	20 (18.2 %)	0.48
Sulfonylureas	13 (11.8%)	11 (10%)	0.48
α– glucosidase inhibitors	3 (2.7%)	1 (0.9%)	0.16
Thiazolidinediones	2 (1.8%)	0 (0%)	NA
ω-3 fatty acids	26 (23.6%)	28 (25.5%)	0.32
Statins	64 (58.2%)	86 (78.1%)	< 0.01
Fibrates	4 (3.6%)	3 (2.7 %)	0.66
ACE-inhibitors	39 (35.5%)	40 (36.3%)	0.61
ARBs	39 (35.5%)	41 (37.3%)	0.08
Calcium channel blockers	46 (41.8%)	53 (48.1%)	0.01
α-blockers	7 (6.4%)	10 (9.1%)	0.18
Urate lowering drugs	36 (32.7%)	51 (46.4%)	< 0.01
Loop-diuretics	54 (49.1%)	59 (53.6%)	0.10
Folic Acid	12 (10.9%)	17 (15.5%)	0.03
Erythropoietin	2 (1.8%)	3 (2.7%)	0.56
Iron supplement	10 (9.1%)	13 (11.8%)	0.18

Data expressed as means ± SD, medians (i.q. range) or proportions (percentage) as appropriate

^a^Paired data Student’s *t* test or McNemar test for continuous or discrete variables, respectively

^b^Log-transformed before performing the parametric analysis. Abbreviations as described in the text

Control group consisted of 101 patients with DKD, aged 72 ± 8 years, 77% of whom were males. With respect to subjects attending the NPD service, control group had comparable median BMI and duration of diabetes, whereas HbA1c levels were slightly but significantly higher [59 (50–64) vs. 54 (47–67) mmol/mol, *p* = 0.005]. No significant differences were observed in LDL-cholesterol, triglycerides, urate and blood pressure levels. A significant lower proportion of patients in the control group had the complete phenotype of DKD (18.2 vs. 38.1%, *p* = 0.004) compared with the NPD group. When comparing ongoing medications between index and follow-up visit in the control group, we registered a significant increase only for SGLT-2 inhibitors prescriptions, whereas no significant differences were seen for GLP-1R agonists or statins prescriptions, as conversely observed in the NPD group. As well, control group patients showed no significant modifications in eGFR, UACR and LDL-C, whereas HbA1c levels significantly ameliorated between index and follow-up visit [59 (50–64) vs. 54 (48–62), *p* = 0.01].

Based on what reported in the medical records of the NPD service, we finally examined the reasons why several patients did not receive prescription of SGLT-2 inhibitors despite their established recommendation for reno-protection. As shown in Table 3, except for those patients with type 1 diabetes or with eGFR below 20 ml/min, the majority of patients were not prescribed with SGLT-2 inhibitors because of medical concerns about concomitant urologic diseases and/or history of urinary infections (more than 50% when taken together).

**Table 3:** Reasons for not prescribing SGLT2-inhibitors at the NPD visit

Diagnosis of type 1 diabetes	2 (4.4%)
Concomitant urologic diseases (urinary incontinence or benign prostatic hyperplasia)	12 (26.7%)
Personal refusal	13 (28.9%)
History of urinary infections	13 (28.9%)
Presence of eGFR < 20 ml/min	5 (11.1%)

Data are proportions (percentage)

## Discussion

Diabetes kidney disease (DKD) is a multi-faceted condition potentially affected by numerous coexisting diseases, including obesity, dyslipidaemia and arterial hypertension. Management of risk factors like high blood pressure and body weight, but also education training of healthcare providers, early detection programs and promotion of evidence-based nephroprotective treatments are all crucial points in order to reduce progression of the renal damage [19]. As such, management of DKD requires a comprehensive and multidisciplinary approach, which can present substantial challenges. Objective of this observational analysis was to evaluate whether an integrated nephrology/diabetology (NPD) approach has the potential to address these challenges by: (1) improving real-time interaction and joint decision-making between specialists; (2) facilitating the exchange of knowledge between nephrologists and diabetologists on the importance of both glycaemic control and protection against renal damage; (3) improving adherence to current treatment guidelines of DKD in terms of evidence-based DKD-specific medications to reduce cardiorenal risk.

According to Table 1, we analyzed a well representative population of patients with DKD, with a median age of 71 years and duration of diabetes of more than 10 years. Most subjects were overweight or obese and had an acceptable glycemic control according to HbA1c, with a median of 7.1% (54 mmol/mol). The only exclusion criterion we applied was being on hemodialysis treatment. Of note, median LDL-cholesterol level was 88 mg/dl, which is above the recommended cut-off level of 70 mg/dl for patients with diabetes and evidence of renal damage [15, 16]. No significant differences were detected among the various DKD phenotypes in terms of glycemic control, BMI, lipid profile and duration of diabetes. Interestingly, only a minority of patients had been previously referred to a nephrologist (23.6% of the population as a whole), even among those with a more aggressive phenotype (35.8% among those with albuminuria and decreased GFR). This is quite surprising since individuals with DKD phenotype characterized by albuminuria and reduced eGFR were reported to have the highest mortality risk over time, compared with diabetic patients with no evidence of renal damage [20]. Moreover, these patients had a long duration of diabetes (more than 10 years) and were already being followed at a specialized diabetology clinic. This point demonstrates the limited awareness among physicians regarding renal disease in diabetes, particularly in its initial stages, and may justify at least in part the under-prescription of drugs with proven renal and cardiovascular benefit in these high-risk patients [12, 13]. Accordingly, before attending the first visit at the NPD outpatient service, only 25.5% and 21.8% of our DKD population was already treated with SGLT-2 inhibitors and/or GLP-1R agonists, respectively (Table 2). At the six-months follow-up visit, prescriptions significantly increased to 54.5% for SGLT-2 inhibitors and 28.3% for GLP-1R agonsts (*p* < 0.05 for both), showing that therapeutic inertia may be counteracted, at least in part, with a simultaneous and integrated multidisciplinary approach [17]. Of note, this increase in prescriptions of anti-hyperglycemic drugs with known reno-protective effects after attending the NPD service, was only partially reported in the control group attending the regular Diabetology outpatient service, only for SGLT-2 inhibitors (*p* < 0.01) but not for GLP-1R agonists (*p* = 0.18). These findings are of concern because may reflect a knowledge gap of diabetologists regarding DKD management and limited consideration of reno-protective effects of GLP-1R agonists [21]. This potential gap of knowledge represents a not negligible obstacle for efficacious clinical management of DKD, since combination therapy with SGLT-2 inhibitors and GLP-1R agonists is currently reimbursed by the Italian Health care System when prescribed by specialists in Diabetology only. From this perspective, the opportunity of a simultaneous and shared evaluation by both a diabetologist and a nephrologist can improve this aspect and provide the patient with the best therapeutic strategy for both optimal glycemic control and kidney damage protection.

Nevertheless, the proportion of patients who had not received a prescription for SGLT-2 inhibitors even after the NPD visit was still significant (approximately 40%). As shown in Table 3, reasons for this lack of prescription were as follows: personal refusal, due to the fear of polyuria and side effects; history of urinary infections, such as recurrent cystitis, genital mycosis or presence of bacteriuria; presence of urologic concomitant disease, such as urinary incontinence and benign prostatic hyperplasia. In a minority of patients (roughly 15%), SGLT-2 inhibitors were not correctly initiated because of presence of type 1 diabetes or eGFR < 20 ml/min. This said, whereas the presence of concomitant urological diseases represents a difficult barrier to overcome for the prescription of this class of drugs, major objective of a shared multidisciplinary approach would be to reduce the number of missed prescriptions due to “personal refusal”, improving confidence of both physicians and patients on the real effectiveness of these drugs in patients with DKD. For instance, it is largely reported that SGLT-2 inhibitors significantly increase the risk of urinary tract infections (UTIs) compared to other glucose-lowering treatments [22]. However, although clinical symptoms such as dysuria, pollakiuria, and urgency were more frequent in patients treated with this class of drugs, these infections were generally mild to moderate in severity and responded well to standard antibiotic treatments. Additionally, the overall risk of severe complications such as urosepsis or pyelonephritis requiring hospitalization remained low with SGLT-2 inhibitors [22]. These findings suggest that with appropriate patient selection and monitoring, benefits of SGLT-2 inhibitors in terms of cardiovascular and renal outcomes clearly outweigh the risks of UTIs [10–11]​. These insights underscore the importance of patient education and regular monitoring for symptoms of UTIs in those receiving SGLT-2 inhibitors, as well as increased confidence in the use of drugs and shared decision making among physicians. Similarly, the upcoming evidence [21] on efficacy of GLP1-R agonists on renal function suggests the possibility of using both class of drugs (GLP-1R agonists and SGLT-2 inhibitors) and the need to share knowledge between diabetologist and nephrologists.

Noteworthy, percentage of patients on statin therapy was 58% prior to the NPD visit, which is relatively low considering the high cardiovascular risk profile of these patients. A number of studies have highlighted the critical role of statins in both protecting against the progression of renal damage and reducing cardiovascular risk in patients with DKD [23]. Beyond their lipid-lowering effects, statins provide significant cardiovascular and renal protective benefits by reducing systemic inflammation, improving endothelial function, and stabilizing atherosclerotic plaques [24]. In accordance, current clinical guidelines highly recommend the use of statins in patients with DKD to reduce cardiovascular risk and protect renal function [25]. For all these reasons, the increased percentage of statins prescriptions after the NPD visit (78.1%, *p* < 0.01) is certainly a positive outcome, further demonstrating the effectiveness of an integrated and collaborative approach to managing patients with DKD, at least in terms of proper treatment guideline application. Similar considerations apply to the prescriptions of urate-lowering drugs, which also significantly increased after attending the NPD visit (from 32.7 to 46.4%, *p* < 0.01). As a matter of fact, high circulating levels of uric acid are considered as a further risk factor for both cardiovascular and renal diseases [26] the effect of urate lowering therapy to protect kidney function and reduce albuminuria in diabetic patients is controversial [27].

Another major risk factor for the progression of CKD is arterial hypertension. Consequently, blood pressure-lowering therapy is a key focus during NPD visits. Managing hypertension in DKD patients is particularly challenging due to the limited use of first-line treatments like RAAS inhibitors and diuretics in patients with reduced eGFR [28]. Given the strict blood pressure targets for patients with diabetes mellitus and the high/very high cardiovascular risk associated with DKD, the ESC/ESH guidelines recommend a first-line treatment approach using a combination of a RAAS inhibitor (either an ACE inhibitor or an ARB) with a calcium channel blocker (CCB) or a diuretic [29]. There is also evidence suggesting that CCBs can reduce proteinuria, cardiovascular disease mortality, and CKD progression [30]. Accordingly, in our study we observed a statistically significant increase in CCBs prescriptions after the NPD visit (from 41.8 to 48.1%, *p* = 0.01), which is likely attributable to a heightened focus on achieving blood pressure targets, leading to the intensification of first-line therapy. In addition to the increase in prescriptions of drugs that are crucial for the management of DKD, the integrated diabetologist/nephrologist approach proposed by the NPD service also produced convincing results on several clinical parameters related to cardio-renal risk. In particular, the significant improvement observed in LDL-C and UACR after six months from the first NPD visit represent a valuable outcome, since both cholesterol levels and urinary albumin excretion have been largely associated with severity and risk of progression of DKD [23]. Of note, these changes were not reported in patients followed by the regular Diabetology outpatient service. As a matter of fact, in these latter patients we observed an adequate focus on glycemic control, as reflected by the significant improvement in HbA1c levels, but not as much attention at reducing the risk of kidney damage progression, in terms of risk factors control and prescriptions of reno-protective drugs, compared with the NPD service.

Finally, it is important to emphasize the general early stage of DKD in our population, with mean eGFR of 48.5 ml/min and mean UACR of 325 mg/g. These patients are those who can probably benefit the most from attending the NPD outpatient service, as there is a therapeutic ‘window’ where the integrated nephrology/diabetology approach can: (I) optimize DKD therapy through the introduction of SGLT-2 inhibitors and/or GLP-1R agonists; (II) assess the individual cardiovascular risk of DKD patients by evaluating and properly managing arterial hypertension, urate levels and lipids. In our view, this represents the major strength of the proposed multidisciplinary integrated approach, in order to address both hesitancy/inertia of nephrologists to prescribe SGLT-2 inhibitors and the suboptimal management of kidney disease by diabetologists, particularly in handling hypertension, hyperuricemia, and dyslipidemia.

Our study has some limitations to acknowledge, mainly related to the reduced sample size and the single-center design. Several clinical parameters, such as nutritional status assessments, were not analyzed since they were not available in the medical records. Similarly, follow-up data are currently limited to six months, so that the observed improvement in clinical parameters need to be confirmed over longer periods of time.

In conclusion, the present study shows that attending an integrated nephrology and diabetology (NPD) outpatient service for patients with type 2 diabetes and concomitant DKD can improve clinical outcomes and increase prescriptions of evidence-based therapies to reduce renal and cardiovascular damage. Additionally, this integrated approach can improve adherence to treatment guidelines for patients with DKD, including recommendations for optimal management of lipid profile and blood pressure.

## Electronic supplementary material

Below is the link to the electronic supplementary material.

Supplementary Material 1

Supplementary Material 2

## Data Availability

Database generated during and/or analyzed during the current study is available from the corresponding author on reasonable request.
