# Supplementary material for: Attending an integrated nephrology and diabetology outpatient service can improve diabetic kidney disease treatment: a single-center experience
Source: Acta Diabetol. 2025 Feb 21;62(7):1057–64. doi: 10.1007/s00592-024-02423-w (PMC12283847; doi:10.1007/s00592-024-02423-w)
Supplement: Supplementary file 2 — Supplementary Material 2 [file 592_2024_2423_MOESM2_ESM.docx]

**Attending an integrated nephrology and diabetology outpatient service can improve diabetic kidney disease treatment: a single-center experience**

Wolde Sellasie S^1,2*^, Pecchioli C^1*^, Cersosimo K^3^, Nardone I^1,2^, Zaccaria S^1,2^, Centi A^3^, Di Perna P^1^, Tatangelo P^3^, Sperti P^1^, Schifano G^5^, Giurato L^1^, Bellia A^4^, Palumbo R^3^ and Uccioli L^1^

^1^Division of Endocrinology and Diabetes, CTO Andrea Alesini Hospital, Department of Biomedicine and Prevention, University of Rome Tor Vergata, 00133 Rome, Italy

^2^PhD School of Applied Medical-Surgical Sciences, University of Rome Tor Vergata, 00133 Rome, Italy

^3^Unit of Nephrology, Sant' Eugenio Hospital, 00144 Rome, Italy

^4^Department of Systems Medicine, University of Rome "Tor Vergata", Rome, Italy.

^5^School of Specialization in Food Sciences, University of Rome Tor Vergata, Via Montpellier 1, 00133, Rome, Italy

**Data availability** Database generated during and/or analyzed during the current study is available from the corresponding author on reasonable request.

**Conflicts of interest** All the authors declare they have no conflict of interest.

**Research involving human participants and/or animals** All procedures performed in this study were in accordance with the ethical standards of the institutional research committee and with the 1964 Helsinki declaration and its later amendments or comparable ethical standards.

**Informed consent** All subjects gave written informed consent for their participation in the study.
