# Supplementary material for: Attending an integrated nephrology and diabetology outpatient service can improve diabetic kidney disease treatment: a single-center experience
Source: Acta Diabetol. 2025 Feb 21;62(7):1057–64. doi: 10.1007/s00592-024-02423-w (PMC12283847; doi:10.1007/s00592-024-02423-w)
Supplement: Supplementary file 1 — Supplementary Material 1 [file 592_2024_2423_MOESM1_ESM.doc]

Rome, July 17**^th^**, 2024

Dear Editor,

Please consider the enclosed manuscript entitled **“Attending an integrated nephrology and diabetology outpatient service can improve diabetic kidney disease treatment: a single-center experience”** for publication in *Acta Diabetologica*.

The research described in this manuscript aimed to explore whether attending an integrated nephrology and diabetology (NPD) outpatient service may improve adherence to treatment guidelines for patients with diabetic kidney disease (DKD). We moved from the observation that, despite the robust evidence supporting efficacy of various new classes of drugs in the management of DKD, optimal care of these patients is not consistently achieved in clinical practice.

For this purpose, we cross-sectionally analyzed patterns of glucose-lowering agents and other DKD-related medications prescription before and after attending the NPD service of our University Hospital in Rome (Italy), in the period between June and November 2023.

We found a significantly increased number of prescriptions of glucose-lowering drugs with proven cardio-renoprotective effects (namely SGLT-2 inhibitors and GLP1-R agonists), as well as for statins, urate-lowering drugs and anti-hypertensive medications (calcium channel blockers).

We think these observations might have several interesting implications, and prompt further analysis of longitudinal data in order to confirm this integrated nephrology/diabetology approach as an innovative model for the outpatient care of subjects with DKD.

Potential reviewers for this manuscript are among:

- **Samy Hadjadj**, Département d’Endocrinologie, Diabétologie et Nutrition, UNIV Nantes, CHU Nantes, Hôpital Guillaume et René Laennec, Nantes, France; samy.hadjadj@univ-nantes.fr
- **Susanna Morano**, Department of Experimental Medicine, “Sapienza” University of Rome, Italy; susanna.morano@uniroma1.it
- **Paola Fioretto**, Department of Medicine (DIMED), University of Padova, Italy; [paola.fioretto@unipd.it](mailto:paola.fioretto@unipd.it)
- **Paolo Menè**, Division of Nephrology, Department of Clinical and Molecular Medicine, “Sapienza” University of Rome, Italy; paolo.mene@uniroma1.it

Finally, we state:

- All the authors have seen and approved this manuscript.
- Neither this manuscript nor any part of this study have been published or are under consideration for publication elsewhere.
- No conflict of interest concerns this manuscript.

We hope that you might find this manuscript of interest and suitable for publication in *Acta Diabetologica*.

Thank you very much for Your consideration.

With best regards.

Sincerely,

Alfonso Bellia, MD, PhD

Associate Professor of Endocrinology

Department of Systems Medicine

University of Rome “Tor Vergata“

Via Montpellier 1

I-00133 Rome, Italy

Phone: +39-06-20902804

E-mail: [bellia@med.uniroma2.it](mailto:bellia@med.uniroma2.it)
